# Proline accumulation and metabolism-related genes expression profiles in *Kosteletzkya virginica* seedlings under salt stress

**DOI:** 10.3389/fpls.2015.00792

**Published:** 2015-09-29

**Authors:** Hongyan Wang, Xiaoli Tang, Honglei Wang, Hong-Bo Shao

**Affiliations:** ^1^Key Laboratory of Coastal Biology and Bioresources Utilization, Yantai Institute of Coastal Zone Research, Chinese Academy of Sciences, YantaiChina; ^2^Yantai Academy of China Agricultural University, YantaiChina; ^3^University of Chinese Academy of Sciences, BeijingChina; ^4^Institute of Agro-biotechnology, Jiangsu Academy of Agricultural Sciences, NanjingChina

**Keywords:** *Kosteletzkya virginica*, salt stress, proline metabolism, proline accumulation, expression profiles

## Abstract

Proline accumulation is a common response to salt stress in many plants. Salt stress also increased proline concentration in roots, stems, and leaves of *Kosteletzkya virginica* seedling treated with 300 mM NaCl for 24 h and reached 3.75-, 4.76-, and 6.83-fold higher than controls. Further study on proline content in leaves under salt stress showed that proline content increased with increasing NaCl concentrations or time. The proline level peaked at 300 mM NaCl for 24 h and reached more than sixfold higher than control, but at 400 mM NaCl for 24 h proline content fell back slightly along with wilting symptom. To explore the cause behind proline accumulation, we first cloned full length genes related to proline metabolism including *KvP5CS1*, *KvOAT*, *KvPDH*, and *KvProT* from *K. virginica* and investigated their expression profiles. The results revealed that the expressions of *KvP5CS1* and *KvProT* were sharply up-regulated by salt stress and the expression of *KvOAT* showed a slight increase with increasing salt concentrations or time, while the expression of *KvPDH* was not changed much and slightly decreased before 12 h and then returned to the original level. As the key enzyme genes for proline biosynthesis, the up-regulated expression of *KvP5CS1* played a more important role than *KvOAT* for proline accumulation in leaves under salt stress. The low expression of *KvPDH* for proline catabolism also made a contribution to proline accumulation before 12 h.

## Introduction

Soil salination has become one of the major abiotic tresses limiting crop growth and productivity all over the world. Saline soil is still rapidly expanding due to the deterioration of global climate and many human activities such as developing land unreasonably, improper irrigation and industrial pollution. The decreasing arable land cannot satisfy the demand of the increasing world population, which is expected to reach about 9.1 billion by 2050, about 34% higher than today’s population. To cope with the serious challenge, there is an urgent need to identify and utilize salt-tolerant plants, which can be used to reclaim salt-affected soils and improve the salt tolerance of crops, or be directly domesticated into crops.

Salinity stress damages plant development by adversely affecting a series of biochemical and physiological processes such as photosynthesis, antioxidant metabolism, mineral nutrients homeostasis, osmolytes accumulation, and hormonal signaling (Misra and Gupta, 2005; Khan et al., 2012). Correspondingly, plants have evolved complex physiological and molecular mechanisms to endure and defend themselves from these adverse environments (Yamaguchi- Shinozaki and Shinoaki, 2006). As one of the most common responses to abiotic stresses, the accumulation of cellular osmolytes has been widely confirmed in many plants. Among many osmolytes, proline is the most widely accumulated compound in plants under stress conditions and it has attracted a lot of studies. The role of proline and its metabolism under stress conditions have received considerable attention in many plants, and now it is generally accepted that proline has multifunctional roles. In addition to functioning as a compatible osmolyte, it can also contribute to scavenging reactive oxygen species (ROS), stabilizing subcellular structures, modulating cell redox homeostasis, supplying energy, and functioning as a signaling molecule to interact with other metabolic pathways under stress conditions (Kavi-Kishor et al., 2005; Verbruggen and Hermans, 2008; Szabados and Savouré, 2010; Sharma et al., 2011). So it is of great importance to understand and utilize the regulatory mechanism of proline metabolism to improve the stress resistance of plants.

In higher plants, there are two pathways involved in proline biosynthesis: the glutamate (Glu) and ornithine (Orn) pathways (Hu et al., 1992; Roosens et al., 1998). The Glu pathway normally lies in the cytosol and chloroplasts. Glu is reduced to glutamate-semialdehyde (GSA) by Δ^1^-pyrroline-5-carboxylate synthetase (P5CS), and spontaneously converted to Δ^1^-pyrroline-5-carboxylate (P5C). P5C is then reduced to proline by Δ^1^-pyrroline-5-carboxylate reductase (P5CR). The Orn pathway occurs in mitochondria. Orn is transaminated to P5C by ornithine-δ-aminotransferase (δ-OAT) and P5C is then transported to the cytosol and converted to proline by P5CR. The Glu pathway generally occurs under stress conditions while the Orn pathway is involved in seedling development (Armengaud et al., 2004). Proline degradation takes place in mitochondria through the sequential actions of proline dehydrogenase (PDH) and Δ^1^-pyrroline-5-carboxylate dehydrogenase (P5CDH), which produce P5C and Glu, respectively. Among the above-mentioned enzymes involved in proline metabolism, P5CS is generally considered to be the key enzyme for proline synthesis while PDH plays a key role in degradation. The enhanced synthesis and decreased degradation of proline are supposed to result in proline accumulation under stress (Chaitanya et al., 2009; Sharma et al., 2011). Thus genetic manipulation of the key enzyme genes by overexpressing *P5CS* gene or suppressing *PDH* gene expression has been widely studied in model plant *Arabidopsis thaliana*, tobacco as well as other plants (Ribarits et al., 2007; Parida et al., 2008; Miller et al., 2009; Sharma et al., 2011). These studies all reveal that there is a close correlation between proline accumulation and stress tolerance (Saradhi et al., 1995; Hare et al., 1999; Siripornadulsil et al., 2002; Kavi-Kishor et al., 2005; Mizoi and Yamaguchi-Shinozaki, 2013). In addition, because of the subcellular compartmentation of proline metabolism in plants, the dynamic transport process of proline is also vital for the protective role of proline, but it is not well understood until now. Although some specific proline transporters have been isolated and characterized, there are only few reports showing a direct role of ProTs in proline transport in plants (Kavi-Kishor et al., 2005; Lehmann et al., 2010; Kavi-Kishor and Sreenivasulu, 2014). A lot of efforts are needed to reveal the roles of proline transport and identify the proline transport systems in the future.

*Kosteletzkya virginica* (L.) is a perennial facultative halophytic species in Malvaceae family, natively distributing in coastal areas from Long Island along the Atlantic coast of the US west to eastern Texas, and is also found in coastal areas of Eurasia (Gallagher, 1985; Blanchard, 2008). It grows frequently in seashore soil containing 0.3–2.5% sodium salt (mainly NaCl; Zhou et al., 2010). Because of its economic values and the tolerance to saline soil, this species has been introduced in China and recommended as a potential cash crop for alternative saline agriculture (Gallagher, 1995). Many studies demonstrate that it is indeed a halophytic plant and is able to act as a model for the exploration of plant resistance. More importantly it is also a genetic resource to serve for our salt-tolerant crops breeding (Tang et al., 2015). Previously, we have studied its salt tolerance physiological characteristics and the results show that salt stress drastically increases proline accumulation, especially under severe salt stress (Wang et al., 2015). However, the specific mechanism of proline accumulation in *K. virginica* has not been reported so far. It is well known that proline level in plants is a combination result of biosynthesis, catabolism and transport processes and this prompts us to clone proline metabolism-related genes from *K. virginica* and further investigate their expression profiles under salt stress. Here we report the results of our experiment.

## Materials and Methods

### Plant Materials and Growth Conditions

The seeds of *K. virginica* were collected from Yellow River Delta, Shandong Province, China. The seeds were soaked in concentrated sulfuric acid for 20 min to remove the hard shell and then thoroughly rinsed with deionized water. The treated seeds were sown in plastic pots (with drain holes in bottom) containing washed sand and grown in the artificial climatic chambers (Huier, China) with temperature of 25°C/20°C(day/night), photoperiod of 16 h/8 h (light/dark) and relative air humidity of 60%. Seedlings were sufficiently watered with 1/2 Hoagland nutrient solution every 3 days. Six seedlings of uniform growth were kept in each pot.

### Stress Treatments and Sampling

Two-week-old seedlings were treated with salt stress. To analyze organ-specific distribution of proline, roots, stems, and leaves were sampled in the unstressed condition and at 24 h after salt stress (300 mM NaCl), respectively. For salt concentration gradient treatments, seedlings were irrigated with 1/2 Hoagland nutrient solution containing different concentrations of NaCl (100, 200, 300, or 400 mM) for 24 h. For the time-course treatments, seedlings were irrigated with 1/2 Hoagland nutrient solution containing 300 mM NaCl for 2, 6, 12, and 24 h treatments. Seedlings with only 1/2 Hoagland nutrient solution treatments were used as controls. Each treatment had three pot replications and the sample from each pot was mixed together as a replication. All samples were snap-frozen in liquid nitrogen and stored at -80°C until use.

### RNA Extraction and First-strand cDNA Synthesis

Total RNA was extracted according to the manufacturer’s instructions of RNAiso Plus (TaKaRa, Japan). The quality and quantity of total RNA were measured by using a NanoDrop-2000c spectrophotometer (Thermo Fisher Scientific, USA). The first-strand cDNA was synthesized with TransScript All-in-One First-Strand cDNA Synthesis SuperMix for PCR (Transgen, China) according to the manufacturer’s instructions.

### Cloning of Proline Metabolism-related Genes

The first strand cDNA synthesized from mixed RNA samples treated by salt stress was used as the template for PCR amplification. According to the conserved region of each gene, a pair of primers (sequences given in Supplementary Table S1) were designed and used to amplify the core fragment of *P5CS*, *OAT*, *PDH*, and *ProT*, respectively. The amplified fragments were ligated into the *pEASY*-Blunt Zero Cloning Vector (TransGen, China) and sequenced. After the fragments were confirmed to be part of candidate genes by Blast analysis, the 5′ and 3′ ends of the full-length cDNA were further amplified according to the instruction of SMART^TM^ RACE cDNA Amplification Kit (Clotech, USA). Gene-specific primers and nested primers of each target gene were designed according to the obtained cDNA fragments (sequences given in Supplementary Table S2). The PCR products were separated by 1% agarose gel electrophoresis and the desired bands with predicted size were excised from the gels and purified, then ligated into the *pEASY*-Blunt Zero Cloning Vector (TransGen, China) and sequenced. Finally, the above obtained three sequences were spliced and assembled into the full length cDNA for each gene by DNAMAN software.

### Bioinformatic Analysis

The nucleotide sequence and the deduced amino acid sequence were analyzed using the DNAMAN software and the BLAST software online^1^. ProtParam software^2^ was used to analyze the basic characteristics of the encoded proteins. TMHMM software^3^ was used to predict transmembrane domains in the encoded proteins. Conserved domains were analyzed using CDD of NCBI^4^. Multiple peptide alignments and phylogenetic analysis were carried out using Clustal X 2.1 and MEGA 5 programs, respectively.

### Determination of Proline Content

Fresh samples for the determination of proline content were the same as those for gene expression analysis. Each treatment had three pot replications and the sample from each pot was mixed together as a replication. Free proline content was determined by ninhydrin assay at A520 nm according to the method described by Bates et al. (1973).

### Expression Analysis of Isolated Genes

Quantitative real-time PCR (qRT-PCR) was performed on an ABI fast 7500 Sequence Detection System (Applied Biosystems, USA) according to the manufacturer’s instructions. The gene EF-1αcloned in our previous study from *K. virginica* was used as the reference gene to normalize the amount of cDNA in each reaction. The qRT-PCR amplifications were carried out in triplicate in a total volume of 20 μL according to the manufacturer’s instructions of SYBR^®^Green Realtime PCR Master Mix (Applied Biosystems). The qRT-PCR program was holding stage, 50°C for 20 s and 95°C for 10 min, followed by 40 cycles of 95°C for 15 s, 60°C for 1 min, and melt curve stage, 95°C for 15 s, 60°C for 1 min, 95°C for 30 s, and 60°C for 15 s. All analyses were based on the *C*_T_ values of the PCR products. The amplification specificity was determined by analyzing the dissociation curves. Experiments were repeated three times and the *C*_T_ values of the triplicate PCRs were averaged and used for the quantification of transcript levels. The quantification of the relative expression levels was performed using the 2^-ΔΔCT^ method (Livak and Schmittgen, 2001). Primer sequences for expression analysis are listed in Supplementary Table S3.

### Statistical Analysis

Data was analyzed by Microsoft Excel 2007 and SPSS 16.0. Mean and standard error (SD) values of three replications were calculated. Data was compared with the control or among treatments by analysis of variance (ANOVA) to discriminate significant differences at *P* ≤ 0.05 followed by least significant difference tests (LSD). Figures were created using Origin 7.5.

## Results

### Isolation and Characterization of Proline Metabolism-related Genes

The transcriptome information of *K. virginica* seedlings with or without salt stress has been established through high-throughout sequencing technology in our laboratory, which was deposited at GenBank with the accession number GCJL00000000 (Tang et al., 2015). Based on the transcriptome information, four cDNA fragments of proline metabolism-related genes including *P5CS*, *OAT*, *PDH*, and *ProT* were obtained. Blast analysis showed these fragments shared significant homologies with genes in the databases of NCBI. Then, by means of RT-PCR and RACE, the full-length cDNAs of *P5CS*, *OAT*, *PDH*, and *ProT* genes were isolated from *K. virginica* and designated as *KvP5CS1*, *KvOAT*, *KvPDH*, and *KvProT*, respectively. The GenBank Accession numbers and their basic characteristics were listed in **Table 1**. The deduced protein sequences are characterized by the basic features such as the number of amino acid residues, molecular weight and isoelectric point (pI), which were shown in **Table 2**.

**Table 1:** Isolated genes related to proline metabolism in *Kosteletzkya virginica*.

Gene name	GenBank accession	Full length of cDNA (bp)	5′-UTR (bp)	ORF length (bp)	3′-UTR (bp)
*KvP5CS1*	KR029088	2420	90	2139	191
*KvOAT*	KR029089	1738	137	1434	167
*KvPDH*	KR029090	1781	47	1560	174
*KvProT*	KR029091	1654	48	1326	280

UTR, untranslated regions; ORF, open reading frame.

**Table 2:** The basic features of protein sequences encoded by isolated genes.

Protein name	Number of amino acid residues	Molecular weight (kDa)	Isoelectric point
KvP5CS1	712	76.894	6.61
KvOAT	477	52.167	8.32
KvPDH	519	57.031	7.64
KvProT	441	48.256	9.35

### Bioinformatics Analysis of Four Proline Metabolism-related Genes

Blast analysis and multiple sequence alignments revealed that these isolated genes had high homology with known genes in GenBank involved in proline metabolism. The deduced amino acid sequence of KvP5CS1 was more than 80% identical to those homologues in GenBank and shared the highest identity of 89% with P5CS in *Theobroma cacao.* As shown in Supplementary Figure S1, it had the same conserved domains as other species, such as ATP and NADPH binding sites, γ-GK and GSA-DH domains (Savouré et al., 1995; Su et al., 2011). The putative KvOAT protein also shared a very high identity with OATs from other plants and had the highest identity of 90% with OAT in *T. cacao* (Supplementary Figure S2). The putative KvPDH and KvProT proteins had lower identity with their homologues and shared 86 and 83% identity with *Gossypium hirsutum* (AFV28788.1) and *T. cacao* (XP_007009121.1), respectively. The prediction of transmembrane domains showed that KvP5CS1, KvOAT, and KvPDH proteins did not contain transmembrane domains (Supplementary Figures S3A–C), while 11 transmembrane domains were found in KvProT protein (Supplementary Figure S3D).

**FIGURE 1:**
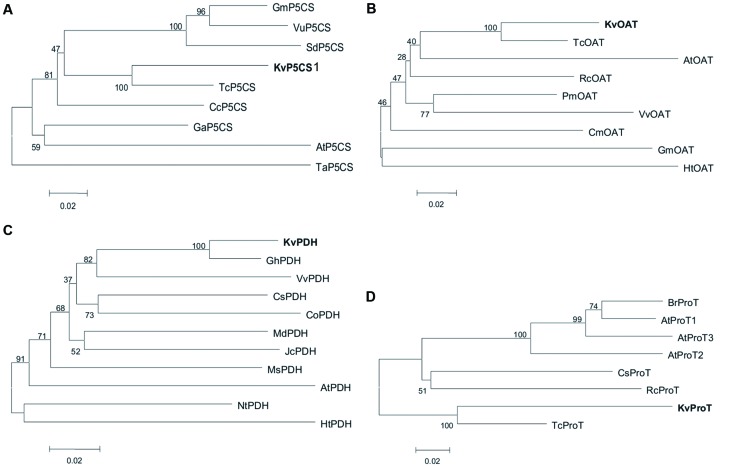
**The phylogenetic analysis for KvP5CS1 **(A)**, KvOAT **(B)**, KvPDH **(C)**, and KvProT (D).** Protein sequences used in phylogenetic analysis were listed in Supplementary Table S4.

Phylogenetic analysis was performed based on multiple protein sequence alignments of different species using a Neighbor-Joining method in the MEGA 5 program. The phylogenetic tree of P5CS proteins from 10 species showed that KvP5CS1 was closely related to *T. cacao* (**Figure 1A**). Both KvOAT and KvProT were also the closest relatives of *T. cacao* in the phylogenetic tree of OAT and ProT, respectively (**Figures 1B,C**), while KvPDH was closely related to *G. hirsutum* (**Figure 1D**).

### Salt Stress Increased Proline Content

**FIGURE 2:**
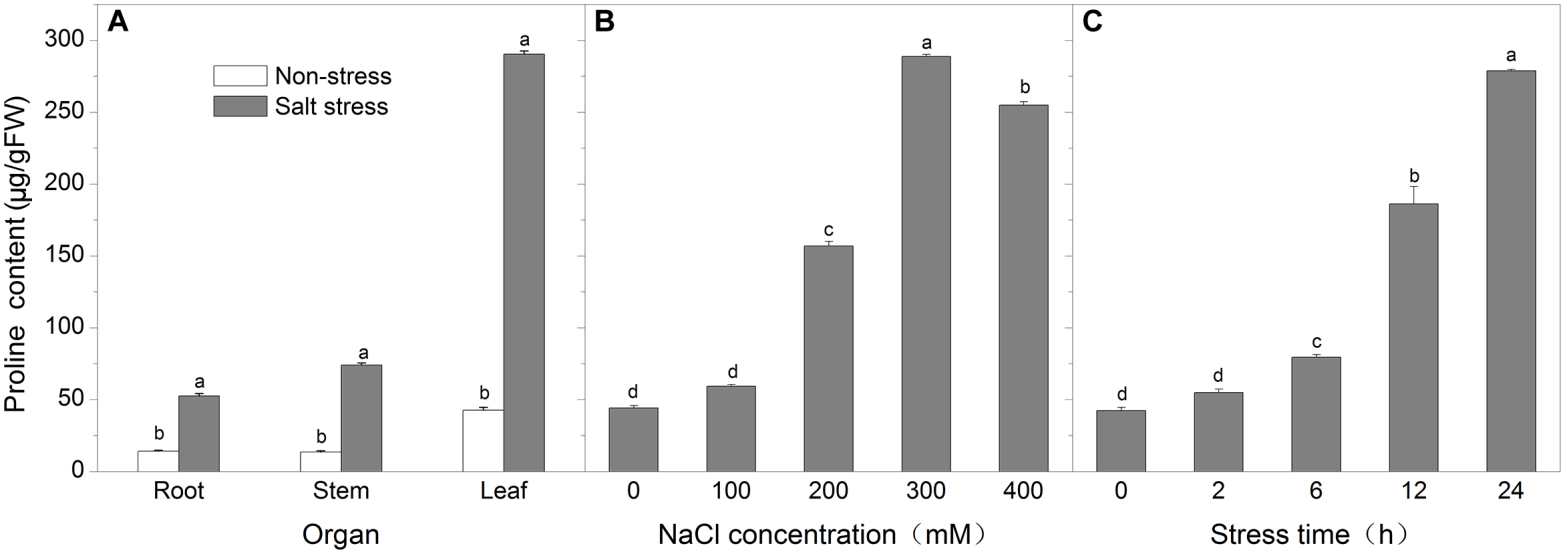
**The changes of free proline content. (A)** The proline content in roots, stems, and leaves under non-stress and 300 mM NaCl for 24 h; **(B)** The proline content in leaves under salt concentration gradient treatments for 24 h; **(C)** The proline content in leaves under time-course treatments with 300 mM NaCl. Each data point represents the average of three replications and the error bars represents standard deviation based on three replications. Data was analyzed by analysis of variance (ANOVA), followed by least significant difference (LSD) tests.

To understand whether proline accumulation is organ specific, we analyzed the proline content in roots, stems and leaves of *K. virginica* seedling treated with 300 mM NaCl for 24 h (**Figure 2A**). Under non-stress conditions, the proline content was the highest in leaves while in roots and stems it was lower. After NaCl stress, the proline content in these three organs all increased remarkably and reached 3.75-, 4.76-, and 6.83-fold higher than the corresponding control, respectively. Obviously, more proline was accumulated in leaves than roots and stems under salt stress. Therefore, the leaves were used in the following experiments.

To find out the correlation between the severity of salt stress and the level of proline accumulation, two-week-old seedlings were treated for 24 h with 0, 100, 200, 300, and 400 mM NaCl and the proline content in leaves was determined. As shown in **Figure 2B**, the proline level peaked at 300 mM NaCl for 24 h and reached more than sixfold higher than control, but at 400 mM NaCl for 24 h proline content fell back slightly along with wilting symptom. So we next chose 300 mM NaCl to treat seedlings for 0, 2, 6, 12, and 24 h in order to investigate the proline accumulation in leaves. The results showed that the proline content gradually increased over time and reached about sixfold higher after 24 h than control (**Figure 2C**).

### Expression Analysis of Four Proline Metabolism-related Genes in Leaves

**FIGURE 3:**
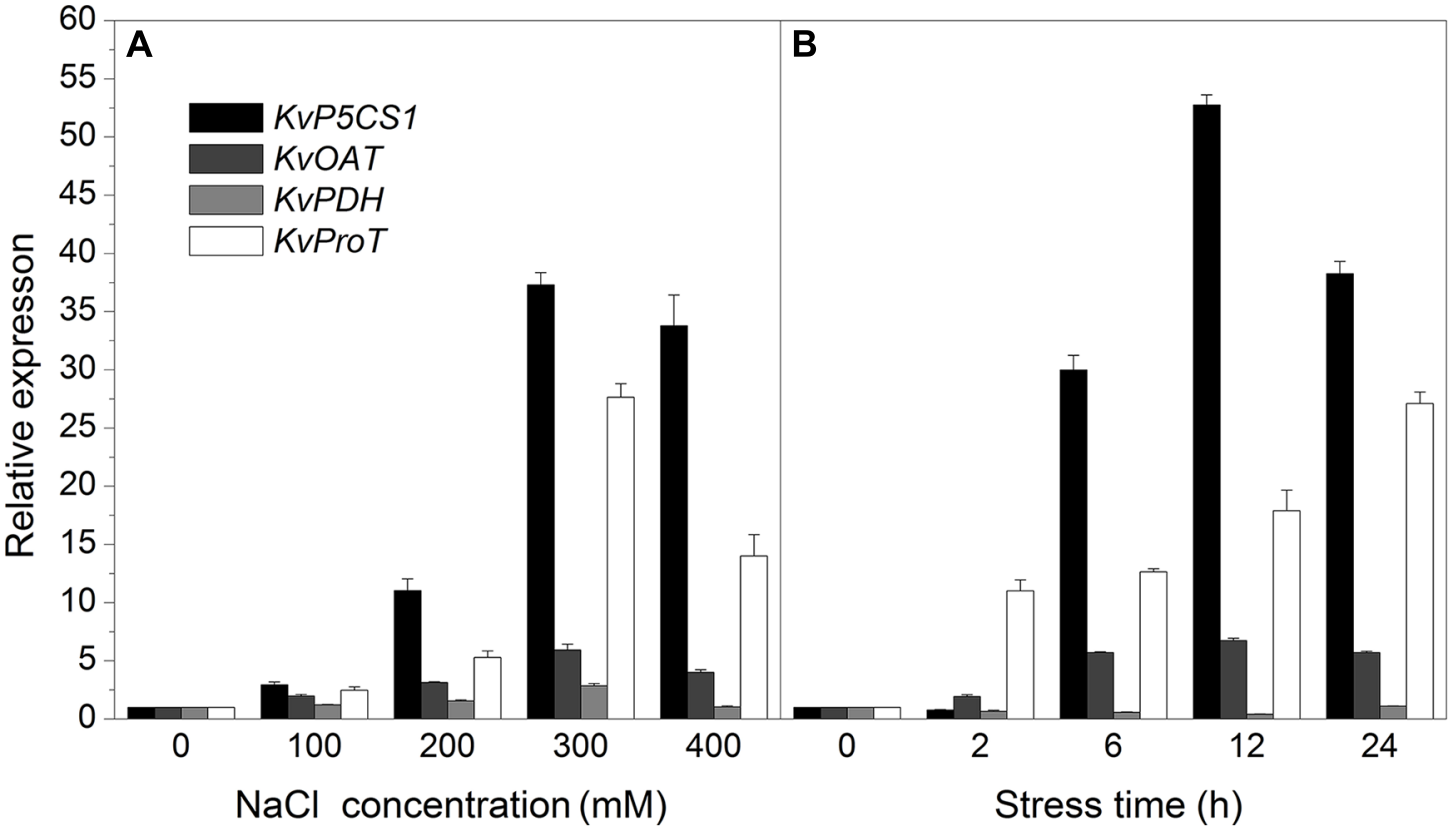
**The expression analysis of *KvP5CS1*, *KvOAT*, *KvPDH*, and *KvProT* in leaves of *Kosteletzkya virginica* under salt stress. (A)** Salt concentration gradient treatments; **(B)** Time-course treatments. Each data point represents the average of three replications and the error bars represents standard deviation based on three replications.

In the experiment of salt concentration gradient treatments (**Figure 3A**), the expression of *KvP5CS1* was slightly increased under low salt stress (100 mM NaCl), and then significantly increased by higher salt stress and peaked at 300 mM NaCl which was approximately 33-fold higher than control, but fell back again slightly at 400 mM NaCl. Expression pattern of *KvProT* showed a similar trend with *KvP5CS1.* The expression of *KvOAT* showed a slight increase with the increasing salt concentrations. The expression of *KvPDH* increased slightly only under 300 mM NaCl stress and under other treatments no significant difference was observed compared to the control.

In view of the obvious effect of 300 mM NaCl on gene expression, we further studied their expression profiles treated with 300 mM NaCl for different time (**Figure 3B**). The expression of *KvP5CS1* was not changed within 2 h and began to increase sharply from 6 to 12 h. The expression of *KvP5CS1* reached the highest level (52.75-fold higher than control) at 12 h and then fell back at 24 h. The expression of *KvOAT* showed a slight increase over time and then stayed at a stable level. The expression of *KvProT* gradually increased over time and reached the highest level at 24 h (27.11-fold higher than control). On the contrary, the expression of *KvPDH* slightly decreased before 12 h and then returned to the original level.

## Discussion

As proline level in plants is a combination result of biosynthesis, catabolism, and transport processes, it is necessary to analyze the expression profiles and functions of genes involved in these processes to understand the mechanism of proline metabolism. So far, there has been no any report about proline-metabolism genes in *K. virginica.* Therefore, we cloned four full-length of cDNAs from *K. virginica-*encoding proline synthetase (KvP5CS1 and KvOAT), proline dehydrogenase (KvPDH), and proline transporter (KvProT). Bioinformatics analysis revealed that the nucleotide and deduced amino sequences of these genes all shared high similarities with the known genes in other plants. For the putative KvP5CS1 protein, it shared the highest similarity (89%) with P5CS in *T. cacao*. Since there were at least two *P5CS* genes characterized in many other plants according to the available reports (Szabados and Savouré, 2010), in the next work we should try to clone another *P5CS* gene from *K. virginica.* KvOAT protein also shared the highest similarity with OAT (90%) in *T. cacao*, while KvPDH was closely related to PDH in *G. hirsutum.* Moreover, the putative KvPDH protein was predicted in the mitochondria by TargetP 1.1 server where proline catabolism regulated by PDH occurs. In contrast, proline synthesis regulated by P5CS and OAT takes place mainly in the cytosol and chloroplasts. As this subcellular compartmentation of proline synthesis and degradation in plants, the dynamic transport of proline is supposed to exist and be of great importance for proline metabolism, especially for stress-induced proline accumulation. In our study, one *KvProT* gene was obtained and its deduced protein shared high similarity with ProT (83%) in *T. cacao* and had 11 transmembrane domains, which indicated that KvProT protein might be located at the mitochondria membrane and involved in the transport of proline. Up to now, some proline transporters have been isolated and characterized in many plants such as *A. thaliana* (Grallath et al., 2005), *Lycopersicon esculentum* (Schwacke et al., 1999), *Oryza sativa* (Igarashi et al., 2000), *Hordeum vulgare* (Fujiwara et al., 2010), and *Chrysanthemum lavandulifolium* (Zhang et al., 2014), but research about their roles in proline transport is still scarce and superficial. It will need a great deal of work to identify the proline transport systems and their functions in plants.

Proline accumulation is a common response to abiotic stress in many plants, but the extent of proline accumulation varies in different plant species. In our study, we firstly analyzed the proline accumulation in different organs of *K. virginica* seedling under salt stress. Like many other plant species, salt stress induced a significant proline accumulation in roots, stems and leaves, especially in leaves where the proline content reached about sixfold higher than control. Furthermore, the proline content in leaves increased with increasing NaCl concentrations and prolonged stress time. The similar finding was reported in green gram (Misra and Gupta, 2005), mulberry (Surabhi et al., 2008), canola (Xue et al., 2009), and *Jerusalem artichoke* (Huang et al., 2013). It has been proposed that leaves accumulate more proline in order to maintain chlorophyll level and cell turgor to protect photosynthetic activity under salt stress (Silva-Ortega et al., 2008).

In previous studies, expression of *Arabidopsis P5CS1* was induced by various types of abiotic stress including salt stress (Savouré et al., 1995; Yoshiba et al., 1995, 1997, 1999; Peng et al., 1996). *P5CS1* overexpression plants increased proline accumulation, while p5cs1 mutants restricted proline accumulation (Kavi-Kishor et al., 1995; Zhang et al., 1995; Szekely et al., 2008). Conversely, reduced expression of *Arabidopsis PDH1* is also thought to be needed for stress-induced proline accumulation (Kiyosue et al., 1996; Yoshiba et al., 1997; Miller et al., 2005; Sharma et al., 2011). These studies on *P5CS1*and *PDH1* in *Arabidopsis* established a “standard model” which meant increased proline synthesis and reduced proline degradation could lead to proline accumulation (Miller et al., 2005, 2009; Ribarits et al., 2007; Parida et al., 2008; Chaitanya et al., 2009; Sharma et al., 2011). Identification of the key genes in proline metabolism from other plant species prompted a wave of studies that sought to overexpress P5CS1 or suppress PDH1expression to increase proline and enhance stress tolerance (Sawahel and Hassan, 2002; Su and Wu, 2004; Tateishi et al., 2005; Molinari et al., 2007). Our results also seemed to fit this model. The expression of *KvP5CS1* was rapidly up-regulated by salt stress, while the expression of *KvPDH* was inhibited before 12 h and only increase slightly by 300 mM NaCl for 24 h. We speculated that inhibited *KvPDH* expression also made a contributed to proline accumulation before 12 h salt stress, while under severe or prolonged salinity, the recovered or increased *KvPDH* expression might speed up the degradation of proline to provide energy and electrons for the respiratory chain (Szabados and Savouré, 2010). As another key enzyme gene for proline synthesis through Orn pathway, *KvOAT* expression showed a slight increase with increasing salt concentrations or prolonged salt stress. Thus, for proline biosynthesis, the up-regulated expression of *KvP5CS1* played a more important role than *KvOAT* for proline accumulation in leaves under salt stress. It is consistent with the findings of previous research that proline biosynthesis from Glu is considered to be the predominant pathway, especially under stress conditions (Armengaud et al., 2004; Lehmann et al., 2010; Szabados and Savouré, 2010). In addition, one *KvProT* gene was isolated and its expression could be obviously up-regulated by salt stress. The increased proline transport might contribute to proline accumulation in leaves from other organs or keep the dynamic balance between proline synthesis and degradation and this still needs to be confirmed by further experiments. Previous studies showed that the expression patterns of different members of the ProT subfamily exhibited organ specificity and disparity under stresses (Rentsch et al., 1996; Igarashi et al., 2000; Fujiwara et al., 2010). In further study, much more attention should be paid to identify other proline transport members and reveal their location as well as their roles in proline transport.

## Conclusion

We cloned four genes related to proline metabolism including *KvP5CS1*, *KvOAT*, *KvPDH*, and *KvProT* from *K. virginica* and investigated their expression profiles in leaves under salinity using quantitative RT-PCR method. The up-regulated expression of *KvP5CS1* and *KvOAT* resulted in proline accumulation in leaves under salt stress, and low expression of *KvPDH* also made a contribution to proline accumulation before 12 h.

## Author Contributions

HW performed the experiments and wrote the manuscript. XT and HW helped with the experimental process. HS revised the paper. All authors reviewed the final manuscript.

## Conflict of Interest Statement

The authors declare that the research was conducted in the absence of any commercial or financial relationships that could be construed as a potential conflict of interest.

## Footnotes

1 http://www.ncbi.nlm.gov/blast

2 http://web.expasy.org/protparam/

3 http://www.cbs.dtu.dk/services/TMHMM/

4 http://www.ncbi.nlm.nih.gov/cdd
