# Supplementary material for: Proline accumulation and metabolism-related genes expression profiles in Kosteletzkya virginica seedlings under salt stress
Source: Front Plant Sci. 2015 Sep 29;6:792. doi: 10.3389/fpls.2015.00792 (PMC4586422; doi:10.3389/fpls.2015.00792)
Supplement: Supplementary file 1 [file Data_Sheet_1.PDF]

Supplementary Material:

Proline accumulation and metabolism-related genes expression profiles in *Kosteletzkya virginica* seedlings under salt stress

Hongyan Wang[1,2,4], Xiaoli Tang[1,4], Honglei Wang[2], Hongbo Shao[1,3*]
*Corresponding author: Prof. Hongbo Shao; e-mail: shaohongbochu@126.com

Supplementary tables

Supplementary table S1. Primers for cloning core fragment of proline metabolism-related genes

Gene	Primer name	Sequence（5’-3’）
P5CS	*P5CS*-F	CCCGGGCGACCCCATGGATCCTTCACG
	P5CS-R	GTCGACAATGCCACACGCAAGTTATG
OAT	*OAT*-F	TGATAAATGGCGGCAACGAGGAAACC
	OAT-R	CACCCATATCTTTCAAGCAAGGCAAC
PDH	*PDH*-F	ATGGCAACAAGAGTTCTCTCATCACCG
	PDH-R	GCATATCAAACCACCCACCGTACCA
ProT	*ProT*-F	GGCAGAAGAGAGGTGAAATT
	ProT-R	AACCGTAACCGAAACGAACA

Note: F forward, R reverse.

Supplementary table S2. Primers for 5’ and 3’ RACE of proline metabolism-related genes

Gene	Primer name	Sequence（5’-3’）
P5CS	*P5CS*-GSP1	CATAAGGCGCTCTTCGAGTGCTGAC
	P5CS-NGSP1	TACCTGAGCCTCTGACGACCAAGACC
	P5CS-GSP2	GTGATGGAGCACGATTCGGACTGGGTG
	P5CS-NGSP2	AGCAGAATCCATGCCCGAGGTCCAGT
OAT	*OAT*- GSP1	TGCTCGGCAAAAACTGGAAACCTGTC
	OAT- NGSP1	ATATGGCTGATCCCTTAGCTTCCGAG
	OAT- GSP2	TCGAGGAAGGGGTTTATTCACGGCTG
	OAT- NGSP2	CCACGCACGACACTATTGTACGGTTG
PDH	*PDH*- GSP1	CCAAAGGTGTTTCGATGAGGCGGGAA
	PDH- NGSP1	AAGCCAATCCGAGGTTTACCGACGAA
	PDH- GSP2	AATGTCGGACGCGCTGTCGTTTGGTT
	PDH- NGSP2	GCCTCGATAGATTACTCATGGGGAAG
ProT	*ProT*- GSP1	CGCGCTTCCTCTAATACCGAACTTGG
	ProT- NGSP1	TGCTGCTCCGTAAGCCCAGTATCCGA
	ProT- GSP2	GTGTCGGCTCTTCTTCCCTTCCTCGG
	ProT- NGSP2	GGTCCAATGTTGTCGTCTTTGCTGTC

Note: GSP gene-specific primer, NGSP nested gene-specific primer.

Supplementary table S3. Primers for expression analysis

Gene	Primer name	Sequence（5’-3’）
P5CS	*P5CS*-qRT-F	CATCAACACGGAAGTTCTCACA
	P5CS-qRT -R	ACTTATTCCAACCTCAGCACCC
OAT	*OAT*-qRT -F	AGGAAGGGGTTTATTCACGGC
	OAT-qRT -R	ATAGTGTCGTGCGTGGGTTTGG
PDH	*PDH*-qRT -F	TTACTTGAAGGACGCCAGGGAG
	PDH-qRT -R	GTGTCTCGGATGCCGTTGTGGA
ProT	*ProT*-qRT -F	CTTCCCTTCCTCGGAGATTTCG
	ProT-qRT -R	GCCAGACCTTTTGTAACAGACT

Note: F forward, R reverse.

Supplementary table S4. Protein sequences used in phylogenetic analysis.

Protein Name		GenBank Accession	Source Organism
P5CS	GmP5CS	XP_003520914.1	*Glycine max*
	VuP5CS	BAI22477.1	*Vigna unguiculata*
	SdP5CS	AFP89589.1	*Sophora davidii*
	TcP5CS	XP_007026911.1	*Theobroma cacao*
	CcP5CS	XP_006429186.1	*Citrus clementina*
	GaP5CS	ACI62865.1	*Gossypium arboreum*
	AtP5CS	CAA60446.1	*Arabidopsis thaliana*
	TaP5CS	BAD97364.1	*Triticum aestivum*
OAT	PmOAT	XP_008225506.1	*Prunus mume*
	VvOAT	NP_001268069.1	*Vitis vinifera*
	TcOAT	XP_007025036.1	*Theobroma cacao*
	AtOAT	NP_199430.1	*Arabidopsis thaliana*
	RcOAT	XP_002519647.1	*Ricinus communis*
	CmOAT	XP_008444491.1	*Cucumis melo*
	HtOAT	AHJ08571.1	*Helianthus tuberosus*
	GmOAT	NP_001237150.1	*Glycine max*
PDH	GhPDH	AFV28788.1	*Gossypium hirsutum*
	VvPDH	XP_002282769.1	*Vitis vinifera*
	CsPDH	XP_006482327.1	*Citrus sinensis*
	CoPDH	AHC08444.1	*Celastrus orbiculatus*
	MdPDH	XP_008377648.1	*Malus domestica*
	JcPDH	AHE13943.1	*Jatropha curcas*
	MsPDH	AAT45084.1	*Medicago sativa*
	AtPDH	NP_189701.3	*Arabidopsis thaliana*
	NtPDH	XP_009611325.1	*Nicotiana tomentosiformis*
	HtPDH	AHJ08572.1	*Helianthus tuberosus*
ProT	BrProT	XP_009143275.1	*Brassica rapa*
	AtProT1	NP_181518.1	*Arabidopsis thaliana*
	AtProT2	NP_191133.1	*Arabidopsis thaliana*
	AtProT3	NP_181198.1	*Arabidopsis thaliana*
	CsProT	XP_004150233.1	*Cucumis sativus*
	RcProT	XP_002530404.1	*Ricinus communis*
	TcProT	XP_007009121.1	*Theobroma cacao*

Supplementary figures and figure legends

Supplementary figure S1. Alignment of KvP5CS1 protein sequences with homologous sequences of other species. The following sequences with corresponding accession number were used for alignment: TcP5CS (XP_007026911.1), CcP5CS (XP_006429186.1), GaP5CS (ACI62865.1), AtP5CS (CAA60446.1), TaP5CS (BAD97364.1). I, ATP binding site; II, putative conserved Leu zipper; III, conserved γ-glutamyl kinase domain; IV, NADPH binding site; V, conserved glutamic- γ -semialdehyde dehydrogenase domain; ↓, phenylalanine site.

Supplementary figure S2. Alignment of KvOAT protein sequences with homologous sequences of other species. The following sequences with corresponding accession number were used for alignment: PmOAT (XP_008225506.1), VvOAT (NP_001268069.1), TcOAT (XP_007025036.1), AtOAT (NP_199430.1), RcOAT (XP_002519647.1), CmOAT (XP_008444491.1), HtOAT (AHJ08571.1), GmOAT (NP_001237150.1).

Supplementary figure S3. Prediction of transmembrane domains in the KvP5CS1, KvOAT, KvPDH and KvProT proteins by TMHMM software. A. KvP5CS; B. KvOAT; C. KvPDH; D. KvProT. Red line represents transmembrane, blue line represents inside and pink line represents outside.

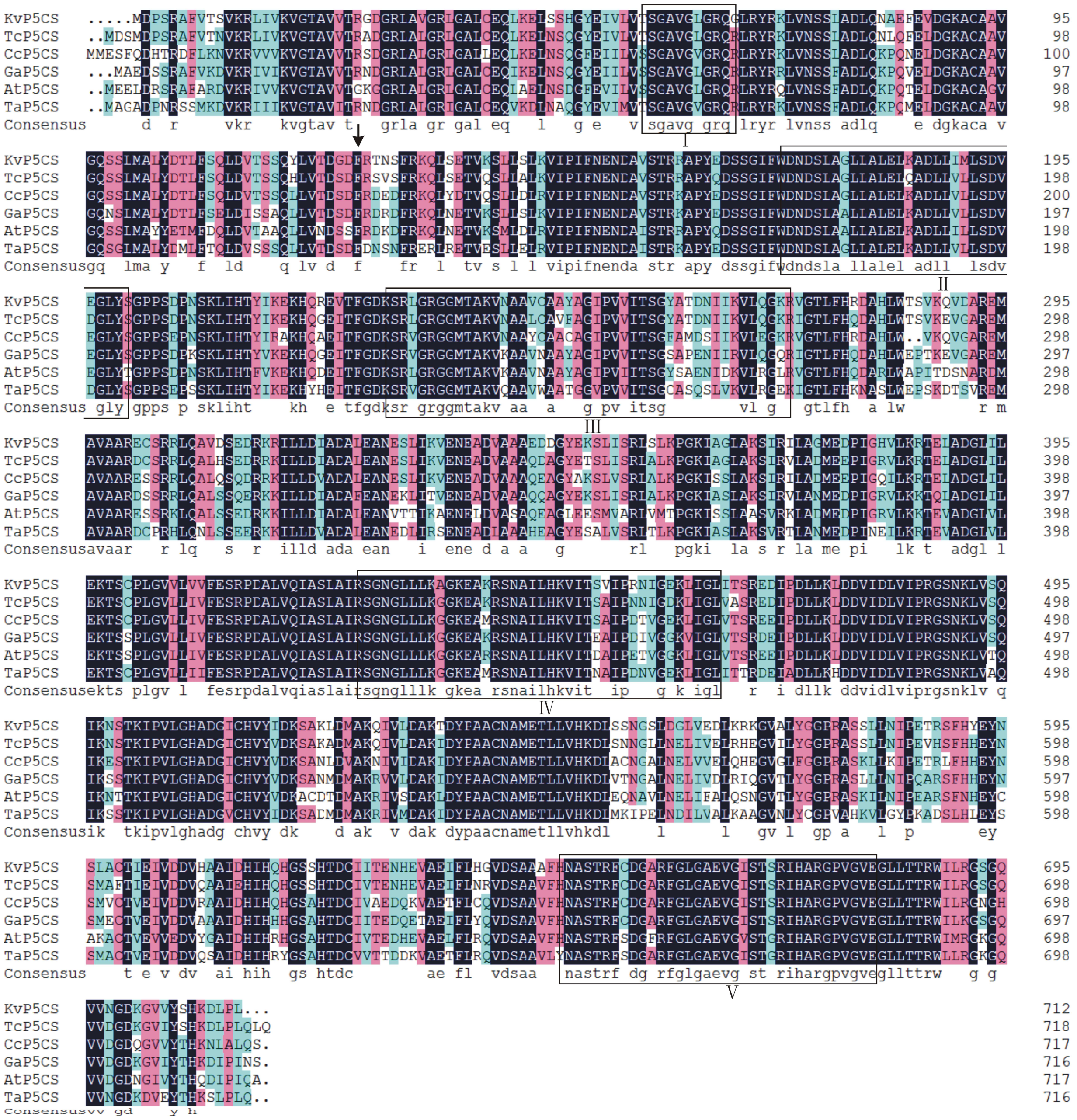

Supplementary figure S1

```
PmOAT    MASRRPLQSVLSSLSLNRVIWSGRRSFGAIPQAT....LPSSSQELISFEYEHSAHNYHPIPVVFSQAKGSSIWDPEGNRYLDFLSAYSAVNQGHCHPKI   96
VvOAT    ........MALATRRFFNTICRGTRSFGALPEG.....IPSSSQHLINLEYEYSAHNYHPIPIVFSQAKGSTIWDPEGKKYLDFLSAYSAVNQGHCHPKV   87
KvOAT    MAATRKPLLSLLT.RVSRTNT..RRAYGVVAEGTSSSSSTSSNHLINLEQEYSAHNYHPVPVVFSEAKGSAIWDPEGKKYLDFLSAYSAVNQGHCHPKI   97
TcOAT    MAATRKPLLSLLS.RVSRAS...RRSYGAVAEG.STSSSPSSSNHLINLEYEYSAHNYHPVPVVFSQAKGSSIWDPEGKKYLDFLSAYSAVNQGHCHPKI   95
AtOAT    MAATTRRLLYYVSKRFSTAGV..RRSYGGLPQS.NSKSPPSSSQRLMELESEFSAHNYHPVPVVFSRANGSTIWDPEGKRYIDFLAAYSAVNQGHCHPKI   97
RcOAT    MASTKRLVQCLLN.RVCRET...RRSYGALPEG....TTSSSSQQLIGLEYDFSAHNYHPVPIVFSRAKGSSIWDPEGNKYLDFLSAYSAVNQGHCHPKI   92
CmOAT    .MASRRQLKCLVS.....LVCRGRRPFSMVPES..SASSFPVSEKLIDMEKDHSAHNYHPIPVVFSEAKGSSIWDPEGKRYLDFLSAYSAVNQGHCHPKI   92
HtOAT    .MASRVPLQLVLN....RIKS..LRCFGTVTEG..IASSTASSRHLIGLEHDYSAHNYHPVPVVFSHGKGSTIWDPEGNKYLDFLAAYSAVNQGHCHPKI   91
GmOAT    .MAATRPVQCLLR.....RVCRGSRTIGVATEVN..ASSSSSSQKIIDKEYEHSAHNYHPLPIVFSQAKGTSVWDPEGNKYLDFLSGYSAVNQGHCHPKI   92
Consensus                        r                    s      e   sahnyhp p vfs   g   wdpeg  y dfl  ysavnqghchpk

PmOAT    MKALQEQAERLTLSSRAFYNDRFPLFAEYLTSMLGYDMVLPMNTGAEAVETALKLARKWGYEKKKIPKNEAIIVSCCGCFHGRTLAVISMSCDNDATRGF  196
VvOAT    LKALVEQAERLTLSSRAFYNDRFPIFAERLMNMFGYDMVLPMNTGAEGVETALKLARKWGYEKKKIPKDQAIIVSCCGLLHGRTLAVISMSCDNEATRGF  187
KvOAT    IKAFQEQAERLTLSSRAFYNDRFPVFAEHLTSMLGYGMVLPMNTGAEGVETALKLARKWGYEKKIIPKDEAIIVSCCGCFHGRTLAVISMSCDNEATRGF  197
TcOAT    TKAFQEQAERLTLSSRAFYNDRFPVFAERLTSMFGYEMVLPMNTGAEGVETALKLARKWGYEKKKIPKDEAIIVSCCGCFHGRTLAVISMSCDNEATRGF  195
AtOAT    MKALQEQVEKLTLSSRAFYNDKFPVFAERLTNMFGYDMVLPMNTGAEGVETALKLARKWGHEKKNIPKDEAIIVSCCGCFHGRTLAIVSMSCDNDATRGF  197
RcOAT    MKALKDQAEKLTLSSRAFYNDRFPLFAERLTSMFGYDMVLPMNTGAEGVETALKLARKWGYEKKKITKDEAIIVSCCGCFHGRTIAVISMSCDNEATRGF  192
CmOAT    VKAFQEQAEKLTLSSRAFYNDKFPLFADYLTQFFGYDMVLPMNTGAEGVETALKLARKWGYKKKRIPKDEAIIVSCCGCFHGRTLGVISMSCDNEATREF  192
HtOAT    MKALIEQAQTLTLSSRAFYNDKFPVFAEYLTKLFGYDMVLPMNTGAEGVETALKLARKWGYEKKRIPKDQAIIVSCCGCFHGRTMGAISMSCDNDATRGF  191
GmOAT    LKALQEQAQRLTVSSRAFYNDRFPVFAEYVTNMFGYDMVLPMNTGAEGVETALKLARKWGYEKKRIPKDEAIIVSCCGCFHGRTLGVISLSCDNEATRGF  192
Consensusks a   q    lt ssrafynd fp fa        gy mvlpmntgae vetalklarkwg  kk i k  aiivsccg  hgrt    s scdn atr f

PmOAT    GPLLPGHLKVDFGDAVGLEKIFEEYGDRIAGFLFEPIQGEAGVIIPPDGYLKSVRELCSKYNILMIADEIQSGLARSGKMLACDWEEVRPDVVILGKALG  296
VvOAT    GPLLPGHLKVDFGDEVALEKIFEENGDRIAGFLFEPIQGEAGVIIPPDGYLKAVRELCSKFNILMIADEIQSGLGRSGRMLACDWAEVRPDVVILGKALG  287
KvOAT    GPLLPGHVKVDFGDAVALEKIFKEHGDKIAGFLFEPIQGEAGVIIPPKGYLKAVRDLCSKYNILMIADEIQSGLARSGKMLACDWEGVRPDIVILGKALG  297
TcOAT    GLLLPGHVKVDFGDDVALEKIFEEHGHRIAGFLFEPIQGEAGVIIPPEGYLKAVRELCSKYNILMIADEIQSGLARTGKMLACDWEEVRPDVVILGKALG  295
AtOAT    GPLLPGNLKVDFGDADSLEKIFKEKGDRIAGFLFEPIQGEAGVIIPPDGYLKAVRELCTKYNVLMIADEVQSGLARSGKMLACDWEEIRPDMVILGKALG  297
RcOAT    GPLLPGHFKVDFGDEVALEKIFKERGDRIAGFLFEPIQGEAGVVIPPDGYLKAVRDLCSKYNVLMIADEIQTGLARTGKMLACDWEQVRPDVVILGKALG  292
CmOAT    GPLLPGHLKVDFGDAVALEKIFIEHGDRIAGFLFEPIQGEAGVIIPPDGYLKAVRDLCTKYNILMIADEIQSGLARSGKLLACDWENVRPDLVILGKALG  292
HtOAT    WPLLPGQLKVDFGDEAALKKVFEEKGEHIAGFLFEPIQGEAGVIIPPDGYLKAVRELCSKHNILMIADEIQSGIARSGRMLACDWEGVRPDVVILGKALG  291
GmOAT    GPLLPGNLKVDFGDAEALERIFKEKGEHIAAFILEPIQGEAGVIFPPDGYLKAVRDICSKYNVLMIADEIQTGLARTGKMLACEWEEVRPDIVILGKALG  292
Consensus llpg  kvdfgd   l   f e g  ia f  epiqgeagv  pp gylk vr   c k n lmiade q g   r g  lac w   rpd vilgkalg

PmOAT    GGVIPVSAVLADKDVMLCIQPGEHGSTFGGNPLASAVAVASLDVIRDERLAERSAQMGQELRNQLLQIQQQYPNYIKEVRGRGLFNAVELNSKNLFPVSA  396
VvOAT    GGVIAVSAVLADKDVMLSIQPGEHGSTFGVNPLASAVAVASLDVITEEGLSERSDQMGQELRNQLMKIHQLFPNLIKEVRGKGLFNAVELNSKALFPVSA  387
KvOAT    GGVIPVSAVLADKDIMLCIRPGEHGSTFGGNPLASAVAIASLDVIQEEKLAERSAHLGEELLRQLVKIKEKFPSYIKEVRGRGLFTAVEFNSKTLFPVSA  397
TcOAT    GGVIPVSAVLADKDVMLCIRPGEHGSTFGGNPLASAVAIASLDVIQEEKLAERSAHLGQELLHQLLKIQEQFPNYIKEVRGRGLFTAVEFNSRTLFPVSA  395
AtOAT    GGVIPVSAVLADKDVMLHIKPGQHGSTFGGNPLASAVAMASLDVIVEEKLVERSASLGEELRIQLNEIKKQFPKYIKEVRGRGLFNAIEFNSESLSPVSA  397
RcOAT    GGVIPVSAVLADKDVMLCIQPGEHGSTFGGNPLASAVAVASLDVVIDEGLAERSAQLGEELRDQLLKVQQQFPDYIKEVRGRGLFNAVEFDSKALSPISA  392
CmOAT    GGVIPVSAVLADKEVMLCIQPGEHGSTFGGNPLASAVAIASLEVIKDERLAERSAKLGEELRNQLIKIQEKFPQYIKNVRGRGLFNAVELKGKALSPVSA  392
HtOAT    GGVIPVSAVLADKDVMLCIQPGEHGSTFRGNPLASAVGIASLQVIQDEKLAERSAEMGEELRQLLTKTQQRFPEIVMEIRGKGLFNAVELTSKSLFPATA  391
GmOAT    GGVIPVSAVLADKDVMLCIQPGQHGSTFGGNPLASAVAIASLEVIKNERLVERSAQMGEELAGQLLKIQQQYPDYVKEVRGRGLFIGVEFNSKNLFPVSG  392
Consensusggvi vsavladk  ml i pg hgstf  nplasav  asl v   e l ers   g el   l        p      rg glf   e     l p

PmOAT    YDICLKLKERGILAKPTHDTIIRLTPPLSMSLGELQEGSKALSDVLELDLPKMQKEKPKNISLDAPNICDRCGRDL.......          472
VvOAT    YDICLKLKERGVLAKPTHDTIIRLTPPLSISLDELQEGSNALRDVMEFDVPKMQKAKHKRVPQTTTNICDRCGRNMDDS....          466
KvOAT    YDICLKMKERGVLAKPTHDTIVRLTPPLCMSPDELKEGSKVLQDVLELDLPNMQKAKP.KDSVKKATICDRCGRNLYDPSD..          477
TcOAT    YDICLKMKERGVLAKPTHDTIVRLTPPLCMSPDELQEGSKVLRDVLELDLPNLQKTKP.KDSPTTATICDRCGRNLYDSSDKD          477
AtOAT    YDICLSLKERGVLAKPTHNTIVRLTPPLSISSDELRDGSEALHDVLELDLPNLLKINSGKTPVSHITECDRCGRNLYA.....          475
RcOAT    YDICLKLKERGILAKPTHDTIVRLTPPLSMSLDELQEGSKALREVLEFDLPNLLKDKPEKASSAASTSCDRCGRNLYASSV..          473
CmOAT    YDICMKLKERGVLAKPTHDTIIRLTPPLSISLDELKEGSKALNDVLEIDLPKLMKEKPHTASSSDSNICDRCGRNLYASED..          473
HtOAT    YDLCIKLKERGILAKPTHNATIRLTPPLSISLDEIREGAKAFHDVLEHDLPKLVKEKPKPASPKARDTCDRCGRELYG.....          469
GmOAT    YELCKKLKYRGVLAKPTHDTIIRFTPPLCISLDEIQQGSKVLADVLEIDLPKLQKTKPKDAAPVASSACDRCGRVLY......          469
Consensusy  c   k rg lakpth     r tppl  s  e   g      v e d p   k             cdrcgr
```

Supplementary figure S2

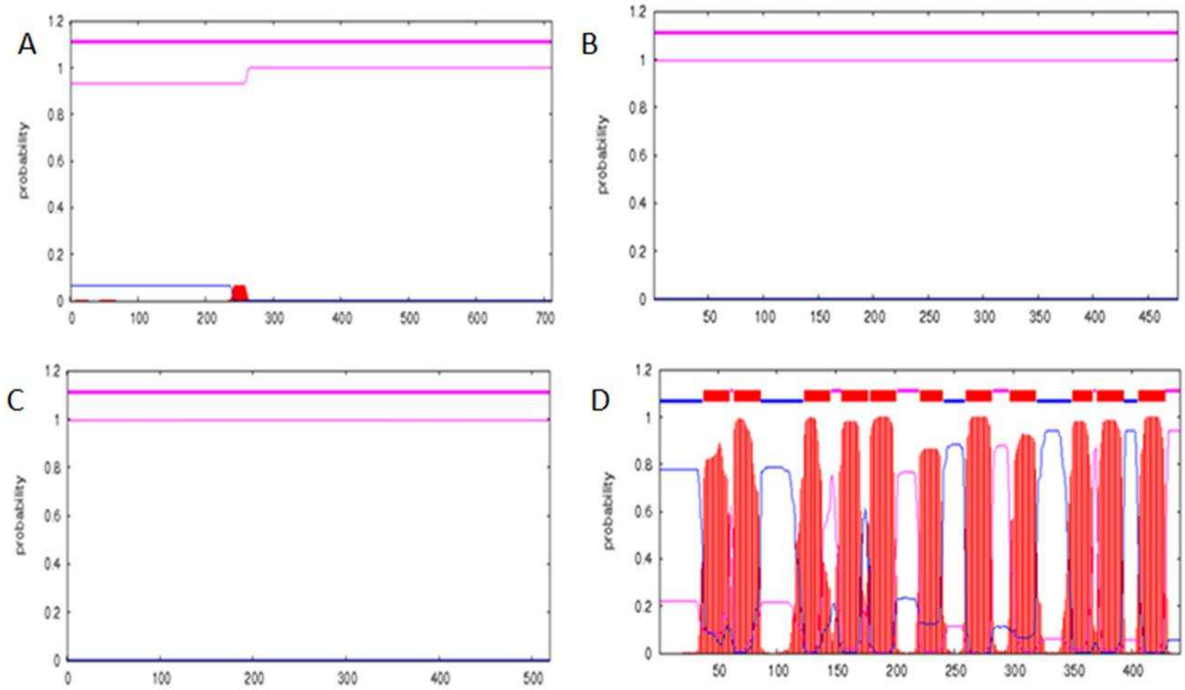

Supplementary figure S3
